# Contrast-Enhanced Ultrasound in the Diagnosis of Gallbladder Diseases: A Multi-Center Experience

**DOI:** 10.1371/journal.pone.0048371

**Published:** 2012-10-31

**Authors:** Lin-Na Liu, Hui-Xiong Xu, Ming-De Lu, Xiao-Yan Xie, Wen-Ping Wang, Bing Hu, Kun Yan, Hong Ding, Shao-Shan Tang, Lin-Xue Qian, Bao-Ming Luo, Yan-Ling Wen

**Affiliations:** 1 Department of Medical Ultrasound, Tenth People’s Hospital of Tongji University, Shanghai, China; 2 Department of Medical Ultrasonics, The First Affiliated Hospital, Sun Yat-Sen University, Guangzhou, China; 3 Department of Ultrasound, Zhongshan Hospital of Fudan University, Shanghai, China; 4 Department of Ultrasound in Medicine, Shanghai Jiao Tong University Affiliated 6th People's Hospital, Shanghai, China; 5 Department of Ultrasound, Peking University School of Oncology, Beijing Cancer Institute, Beijing, China; 6 Department of Ultrasound, Shengjing Hospital of China Medical University, Shenyang, China; 7 Department of Ultrasound, Beijing Friendship Hospital, Capital Medical University, Beijing, China; 8 Department of Ultrasound, Second Affiliated Hospital, Sun Yat-sen University, Guangzhou, China; 9 Department of Ultrasound, Sixth Affiliated Hospital, Sun Yat-sen University, Guangzhou, China; National Cancer Center, Japan

## Abstract

**Objective:**

To assess the usefulness of contrast–enhanced ultrasound (CEUS) in differentiating malignant from benign gallbladder (GB) diseases.

**Methods:**

This study had institutional review board approval. 192 patients with GB diseases from 9 university hospitals were studied. After intravenous bonus injection of a phospholipid-stabilized shell microbubble contrast agent, lesions were scanned with low acoustic power CEUS. A multiple logistic regression analysis was performed to identify diagnostic clues from 17 independent variables that enabled differentiation between malignant and benign GB diseases. Receiver operating characteristic (ROC) curve analysis was performed.

**Results:**

Among the 17 independent variables, multiple logistic regression analysis showed that the following 4 independent variables were associated with the benign nature of the GB diseases, including the patient age, intralesional blood vessel depicted on CEUS, contrast washout time, and wall intactness depicted on CEUS (all *P*<0.05). ROC analysis showed that the patient age, intralesional vessels on CEUS, and the intactness of the GB wall depicted on CEUS yielded an area under the ROC curve (Az) greater than 0.8 in each and Az for the combination of the 4 significant independent variables was 0.915 [95% confidence interval (CI): 0.857–0.974]. The corresponding Az, sensitivity, and specificity for the age were 0.805 (95% CI: 0.746–0.863), 92.2%%, and 59.6%; for the intralesional vessels on CEUS were 0.813 (95% CI: 0.751–0.875), 59.8%, and 98.0%; and for the GB wall intactness were 0.857 (95% CI: 0.786–0.928), 78.4%, and 92.9%. The cut-off values for benign GB diseases were patient age <53.5 yrs, dotted intralesional vessels on CEUS and intact GB wall on CEUS.

**Conclusion:**

CEUS is valuable in differentiating malignant from benign GB diseases. Branched or linear intralesional vessels and destruction of GB wall on CEUS are the CEUS features highly suggestive of GB malignancy and the patient age >53.5 yrs is also a clue for GB malignancy.

## Introduction

Ultrasound (US) examination is accepted as the primary imaging modality in the assessment of gallbladder (GB) disease, which is ascribed to its inherent superiority in comparison to other imaging modalities such as real-time scanning, easy manipulation, cost-effectiveness, no radiation, high resolution, and repeatability. In addition, GB is particularly suitable for US scanning since the echo contrast between GB lesions and the bile in the GB is obvious in most cases, which makes it easy for the delineation of the GB diseases [1], [2], [3], [4].

GB cancer is an uncommon malignancy but highly lethal with a median survival of 6 months, indicating that the majority of patients present with advanced disease. Despite the widespread use of modern imaging techniques, early diagnosis is rare because there are no specific signs and symptoms, and many GB carcinomas are not diagnosed preoperatively. Despite of its obvious superiority, US has difficulty to make definite diagnosis for GB carcinoma under some circumstances [4], [5], [6]. The differential diagnosis includes the more frequently encountered inflammatory conditions of the gallbladder, adenomyomatosis, polyps, biliary sludge, and other hepatobiliary malignancies. Therefore, it is necessary to develop new US techniques to improve the diagnosis of GB diseases and facilitate the early diagnosis of GB carcinoma.

Contrast-enhanced ultrasound (CEUS) under low acoustic power allows clear depiction of macro- and micro- circulation of target organ, which in turn improves the detection and characterization of lesions in various organs such as liver, kidney, pancreas, and so on [7], [8], [9], [10]. Although CEUS has been tentatively used in GB diseases, the role of CEUS in GB is less well recognized and multicenter experience is not available [10], [11], [12], [13], [14], [15], [16]. In this prospective study, the application of GB CEUS in 9 centers was reported with an aim to evaluate the role of CEUS in the differential diagnosis between benign and malignant GB diseases.

**Figure 1:**
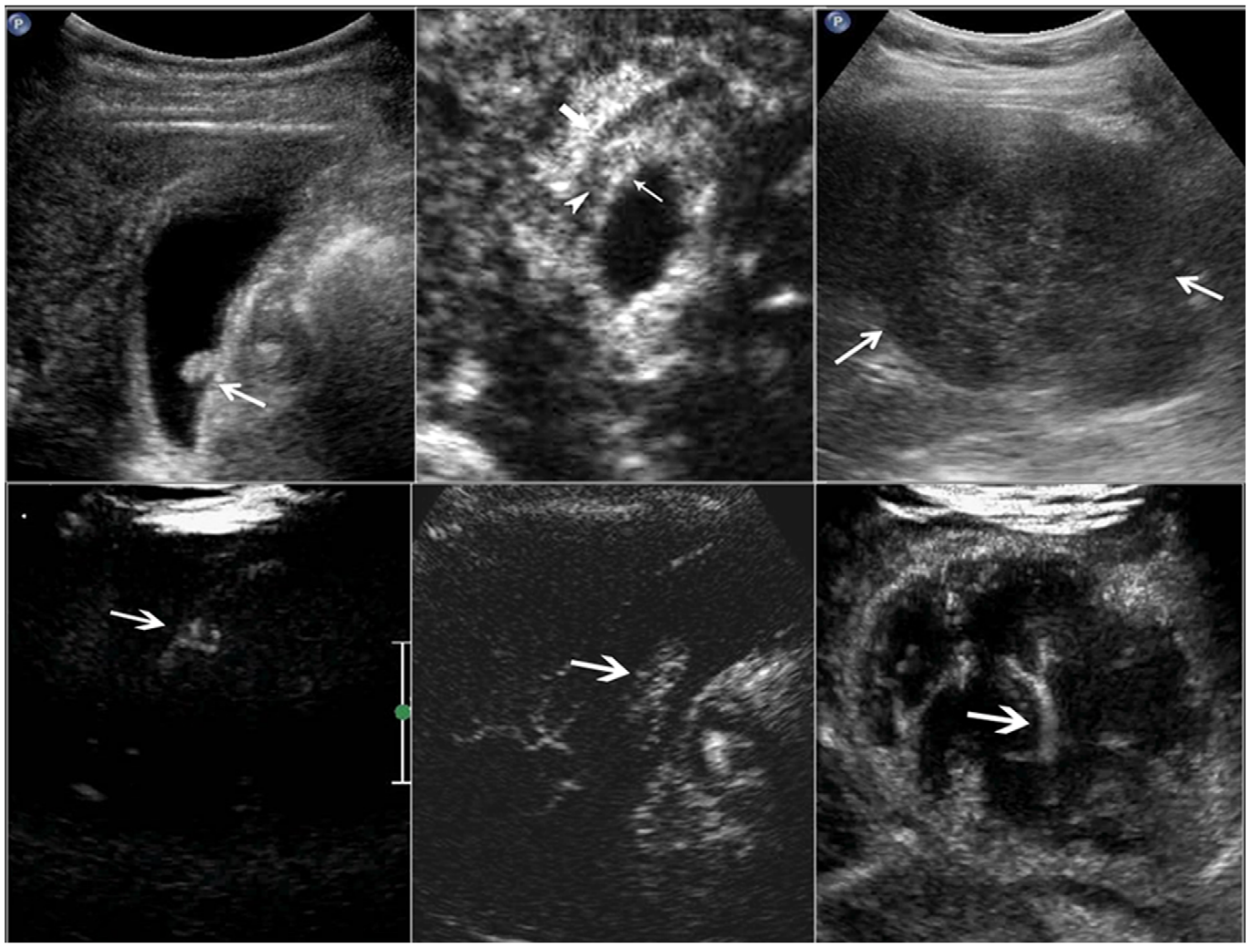
Morphological type and intralesional blood vessels in the gallbladder lesion. Upper left, polypoid type; upper middle, thickened-wall type; upper right, mass-forming type. Lower left, scattered blood vessels; lower middle, linear blood vessels; lower right, branched blood vessels.

## Materials and Methods

### Ethics Statement

This prospective study received approvals from the institutional ethical committees of the 9 university hospitals as follows.

1. Ethical committee of Tenth People’s Hospital of Tongji University.
2. Ethical committee of The First Affiliated Hospital of Sun Yat-Sen University.
3. Ethical committee of Zhongshan Hospital of Fudan University.
4. Ethical committee of Shanghai Jiao Tong University Affiliated 6th People's Hospital.
5. Ethical committee of Peking University School of Oncology, Beijing Cancer Institute.
6. Ethical committee of Shengjing Hospital of China Medical University.
7. Ethical committee of Beijing Friendship Hospital of Capital Medical University.
8. Ethical committee of Second Affiliated Hospital of Sun Yat-sen University.
9. Ethical committee of Sixth Affiliated Hospital of Sun Yat-sen University.

All patients gave written informed consent after the procedure has been carefully explained to the patient. The clinical investigation has been conducted according to the principles expressed in the Declaration of Helsinki.

**Figure 2:**
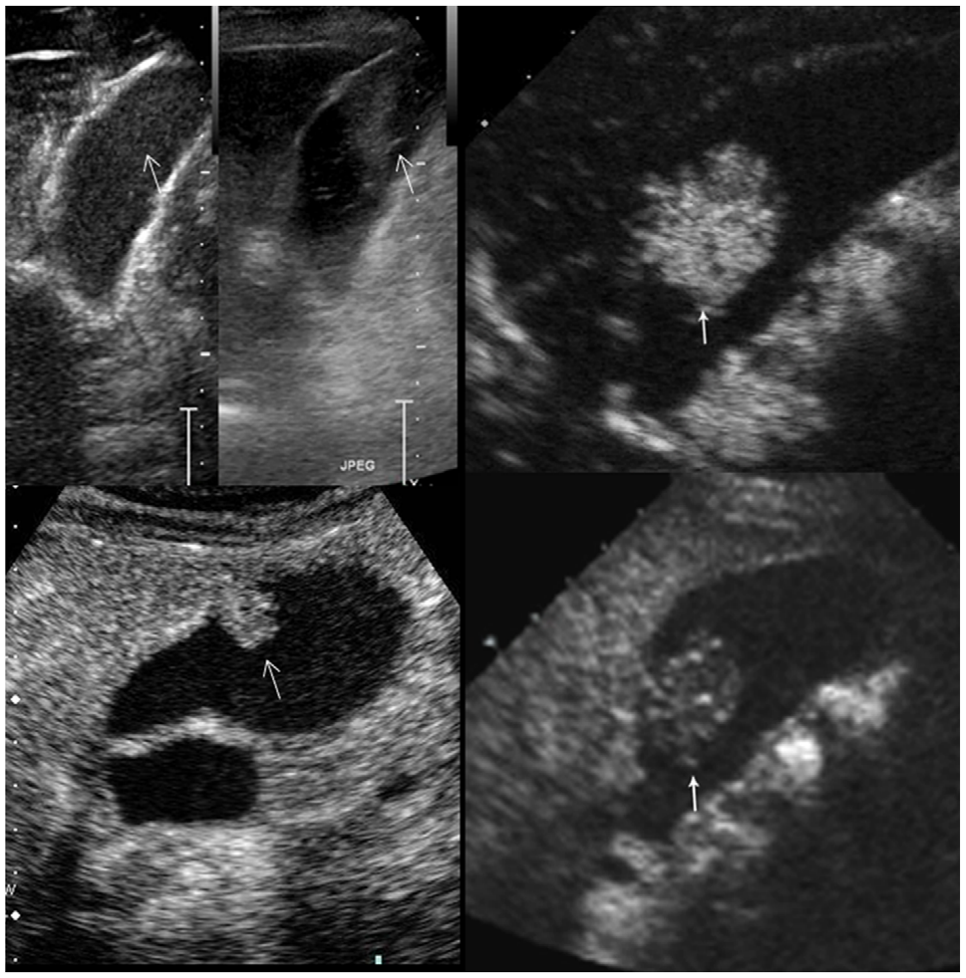
Enhancement extent of the gallbladder lesions on contrast-enhanced ultrasound. Upper left, non-enhancing; upper right, hyper-enhancing; lower left, iso-enhancing; lower-right, hypo-enhancing (arrows indicate the lesions).

**Figure 3:**
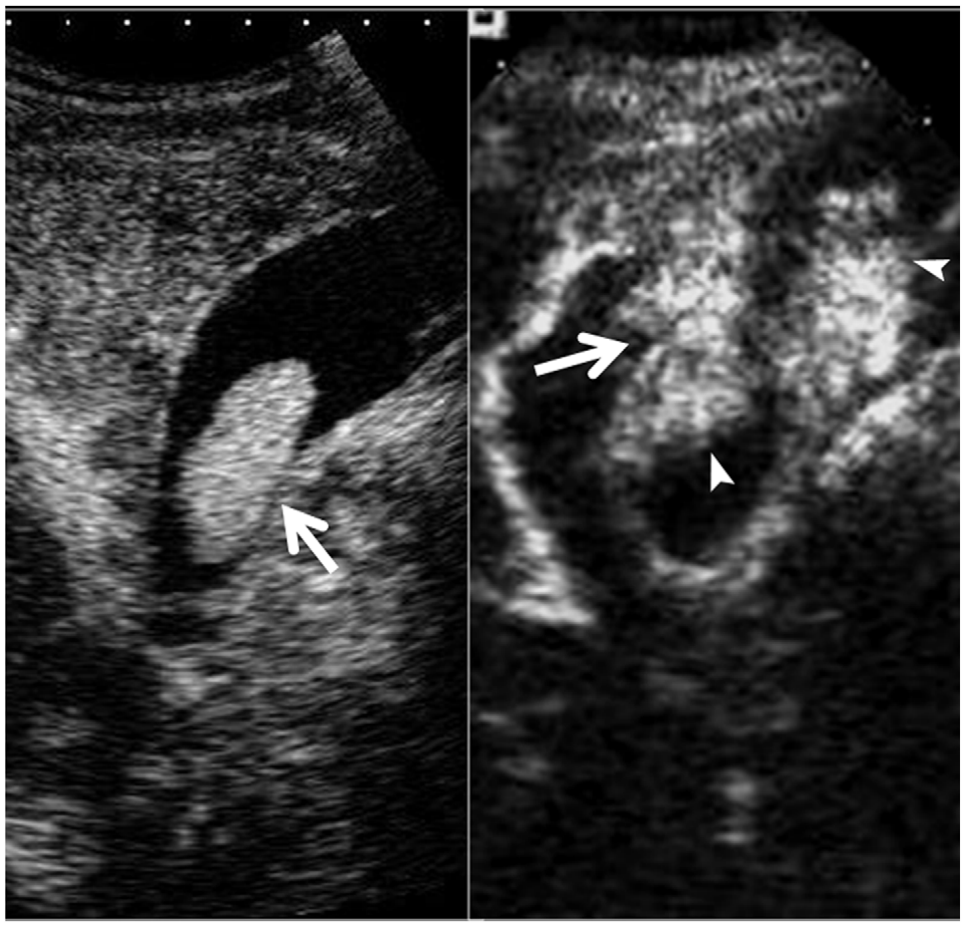
Intactness of the gallbladder wall on contrast-enhanced ultrasound. Left, the gallbladder wall (arrow) beneath an adenoma is intact. Right, the gallbladder wall (arrow) beneath a cancer (arrowheads) is destructed and the infiltration to the adjacent liver (arrow) is observed.

### Study Population

The patients were recruited for CEUS when they met the following criteria: (1) GB diseases found at primary US examinations; (2) Referral for exclusion of malignancy by clinicians or radiologists; (3) Patient age 18 to 80 years old; (4) Absence of severe cardiopulmonary diseases; (5) Not pregnant or lactating; (6) Diagnoses confirmed by pathological examination or surgery. Patients that showed typical US findings of gallbladder stones, debris, and small polypoid lesions (equal to or less than 0.5 cm in diameter) were excluded from the study. Finally, between 2007 and 2010, 192 patients were included in this study. Eight-three (43.2%) of them were male and 109 (56.8%) were female. The age range of the patients was 21 to 80 years old and the mean age (standard deviation, std) was 52 (15) years old. In 161(83.9%) patients, only single lesion was found, whereas in the remaining 31 (16.1%) patients, multiple lesions were detected. In the patients with multiple lesions, generally the largest lesion on US was selected for analysis; however, if the largest lesion was inconspicuous on US, the most conspicuous one of the remaining lesions was selected for analysis.

**Table 1:** Basic characteristics and baseline US characteristics of the patients with gallbladder diseases.

Characteristics	Malignant (n = 51)	Benign (n = 141)						*P* *
		Cholesterol polyp (n = 63)	Adenoma (n = 21)	Adenomyomatosis (n = 14)	Chronic cholecystitis (n = 31)	Biliary sludge (n = 12)	Total (n = 141)	
Patient								
Gender (Male/Female)	25/26	19/44	8/13	9/5	17/14	5/7	58/83	0.410
Age (yrs) mean(std)	63(10)	45(12)	49(18)	46(10)	54(14)	48(17)	48(14)	<0.001
range (min - max)	(28–78)	(24–74)	(21–86)	(26–61)	(25–74)	(26–76)	(21–86)	
Single lesion/Multiple lesions	48/3	41/22	19/2	13/1	31/0	9/3	113/28	0.020
Lesion								
Size (cm) mean (std)	4.5(2.9)	1.1(0.5)	2.3(0.9)	1.2(0.5)	1.6(1.4)	3.7(2.1)	1.7(1.3)	<0.001
range (min - max)	(0.6–14.7)	(0.5–3.3)	(1.2–4.4)	(0.6–2.3)	(0.4–5.8)	(1.0–8.0)	(0.4–14.7)	
≤1.0 cm	3 (5.9%)	36 (57.1%)	0	7(50.0%)	15(48.4%)	1(8.3%)	59(41.8%)	<0.001
1.1–2.0 cm	8 (15.7%)	25 (39.7%)	13(61.9%)	6(42.9%)	8(25.8%)	2(16.7%)	54(38.3%)	
2.1–3.0 cm	6 (11.8%)	1 (1.6%)	3(14.3%)	1(7.1%)	3(9.7%)	2(16.7%)	10(7.1%)	
>3.0 cm	34 (66.7)	1 (1.6%)	5(23.8%)	0	5(16.1%)	7(58.3%)	18(12.8%)	
Location								
Neck	9 (17.6%)	10(15.9%)	2(9.5%)	0	1(3.2%)	0	13(9.2%)	<0.001
Body	10(19.6%)	41(65.1%)	16(76.2%)	7 (50.0%)	7(22.6%)	5(41.7%)	76(53.9%)	
Bottom	10(19.6%)	11(17.5%)	3(14.3%)	4 (28.6%)	5(16.1%)	4(33.3%)	27(19.1%)	
All	22(43.1%)	1(1.6%)	0	3 (21.4%)	18(58.1%)	3(25.0%)	25(17.7%)	
Morphological Type								
Polypoid	8 (15.7%)	63(100%)	20(95.2%)	6(42.9%)	5(16.1%)	6 (50.0%)	100(70.9%)	<0.001
Thickened wall	18(35.3%)	0	0	7(50.0%)	21(67.7%)	0	28(19.9%)	
Mass-forming	25 (49.0%)	0	1(4.8%)	1(7.1%)	5(16.1%)	6 (50.0%)	13(9.2%)	
Echogenicity								
Hyper-	8(15.7%)	48(76.2%)	14(66.7%)	4(28.6%)	8(25.8%)	5(41.7%)	79(56.0%)	<0.001
Iso-	15(29.4%)	9(14.3%)	7(33.3%)	7 (50.0%)	7(22.6%)	4(33.3%)	34(24.1%)	
Hypo-	23(45.1%)	6(9.5%)	0	3(21.4%)	12(38.7%)	3(25.0%)	24(17.0%)	
Mixed	5(9.8%)	0	0	0	4(12.9%)	0	4(2.8%)	
Vascularity								0.001
None	5 (9.8%)	10(15.9%)	2(9.5%)	6(42.9%)	6(19.3%)	11(91.7%)	35(24.8%)	
Scarce	27(52.9%)	49(77.8%)	12(57.1%)	7(50.0%)	18(58.1%)	1(8.3%)	87(61.7%)	
Abundant	18(35.3%)	4(6.3%)	7(33.3%)	1(7.1%)	7(22.6%)	0	19(13.5%)	

* Comparisons between malignant and benign gallbladder diseases.

**Table 2:** Enhancement features of the gallbladder diseases on contrast-enhanced ultrasound.

Enhancement features	Malignant (n = 51)	Benign (n = 141)						*P* *
		Cholesterol polyp (n = 63)	Adenoma (n = 21)	Adenomyomatosis (n = 14)	Chronic cholecystitis (n = 31)	Biliary sludge (n = 12)	Total^#^ (n = 141)	
Enhancement time								
Arrival time (sec)	15.5(3.4)	15.6(3.8)	13.6(3.8)	13.6(3.6)	16.9(4.2)	NA	15.3(4.0)	0.509
range (min - max)	(9–24)	(9–27)	(7–20)	(7–22)	(10–27)		(7–27)	
Time to peak (sec)	20.3(4.4)	18.9(3.8)	18.0(4.3)	18.0(3.7)	20.8(4.0)	NA	19.0(4.0)	0.028
range (min - max)	(10–32)	(13–30)	(11–27)	(13–27)	(14–30)		(11–30)	
Washout time (sec)	41.4(19.4)	47.7(24.2)	87.3(34.7)	53.1(25.5)	62.4(36.1)	NA	58.2(32.2)	<0.001
range (min - max)	(15–120)	(18–120)	(27–170)	(26–120)	(25–176)		(18–176)	
Intralesional vessels								
Branched	6 (11.8%)	0	0	0	0	NA	0	<0.001
Linear	44 (86.3%)	10(15.9%)	17(81.0%)	9(64.3%)	15(48.4%)	NA	51 (39.5%)	
Dotted	1(1.9%)	53(84.1%)	4(19.0%)	5(35.7%)	16(51.6%)	NA	78 (60.5%)	
Enhancement pattern								
Homogeneous	16 (31.4%)	63(100%)	21(100%)	9(64.3%)	17(54.8%)	NA	110 (85.3%)	<0.001
Inhomogeneous	35(68.6%)	0	0	5(35.7%)	14(45.2%)	NA	19 (14.7%)	
Enhancement extent								
Arterial phase								
Hyper-	45(88.2%)	50(79.4%)	20(95.2%)	13(92.9%)	28(90.3%)	NA	111(86.0%)	0.008
Iso-	3(5.9%)	13(20.6%)	1(4.8%)	1(7.1%)	3(9.7%)	NA	18(14.0%)	
Hypo-	3(5.9%)	0	0	0	0	NA	0	
Venous phase								
Hyper-	0	0	0	0	0	NA	0	0.117
Iso-	0	2(3.2%)	1(4.8%)	1(7.1%)	2(6.5%)	NA	6 (4.7%)	
Hypo-	51(100%)	61(96.8%)	20(95.2%)	13(92.9%)	29(93.5%)	NA	123(95.3%)	
Wall intactness								
Intact	11(21.6%)	63(100%)	21(100%)	12(85.7%)	24(77.4%)	12(100%)	132(93.6%)	<0.001
Destructed	40(78.4%)	0	0	2(4.3%)	7(22.6%)	0	9(6.4%)	
Liver infiltration								
Present	26(51.0%)	0	0	0	1(3.2%)	0	1(0.7%)	<0.001
Absent	25(49.0%)	63(100%)	21(100%)	14(100%)	30(96.8%)	12(100%)	140(99.3%)	

* Comparisons between malignant and benign gallbladder diseases. ^#^ n = 129 after excluding the 12 lesions of biliary sludge in the categories of intralesional vessel, enhancement pattern and enhancement extent. NA, not applicable.

### Ultrasound Technique

The following US systems were used: Acuson Sequoia 512 (Contrast pulse sequencing, CPS) (Siemens Medical Solutions, Mountain View, CA), Aplio XV (Contrast harmonic imaging, CHI) (Toshiba Medical System, Tokyo, Japan), IU 22 (Pulse inversion imaging) (Philips Medical Systems, Bothell, Wash); LOGIQ 9 (Coded phase inversion, CPI) (GE Healthcare, Milwaukee, WI); and Esaote DU8 (Contrast tuned imaging, CnTI) (Italy). Since different US systems were used in this multi-center study, the study protocol was standardized prior to implementation. All the transducers were required to be abdominal use and the frequency of the transducers ranged from 1.0 to 6.0 MHz. Contrast specific imaging (CSI) modes were available for all the systems and the range of the mechanical index (MI) for the CSI modes was 0.05 to 0.20, which enables effective tissue cancellation to generate almost pure microbubble images and avoids destruction of microbubbles in the circulation. The machine settings were optimized with the help of the engineers from the manufacturers after review of the study protocol. Uniform imaging setting was used for the same type of system.

**Figure 4:**
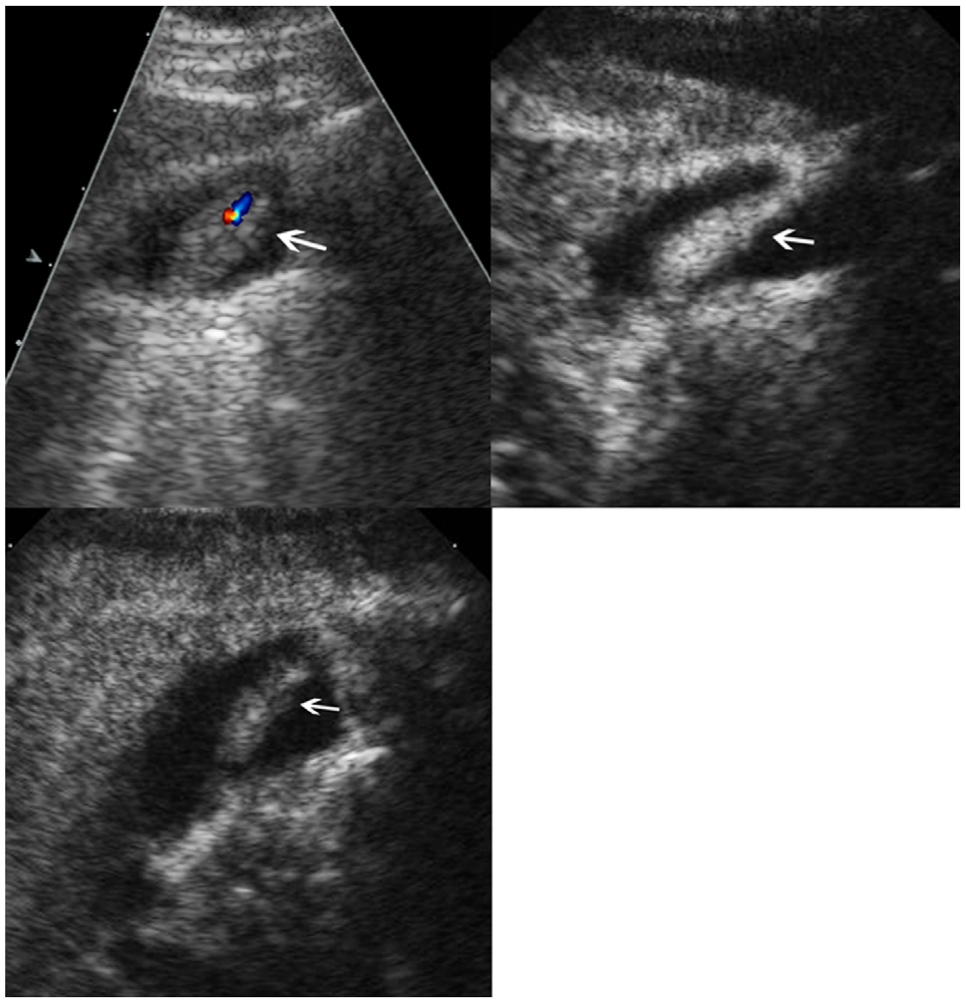
Cholesterol polyp in gallbladder. Upper left, color flow imaging shows intralesional vascularity in the polyp (arrow); Upper right, the lesion (arrow) shows hyper-enhancement during the arterial phase; Lower left, the lesion (arrow) shows iso-enhancement during the venous phase.

**Figure 5:**
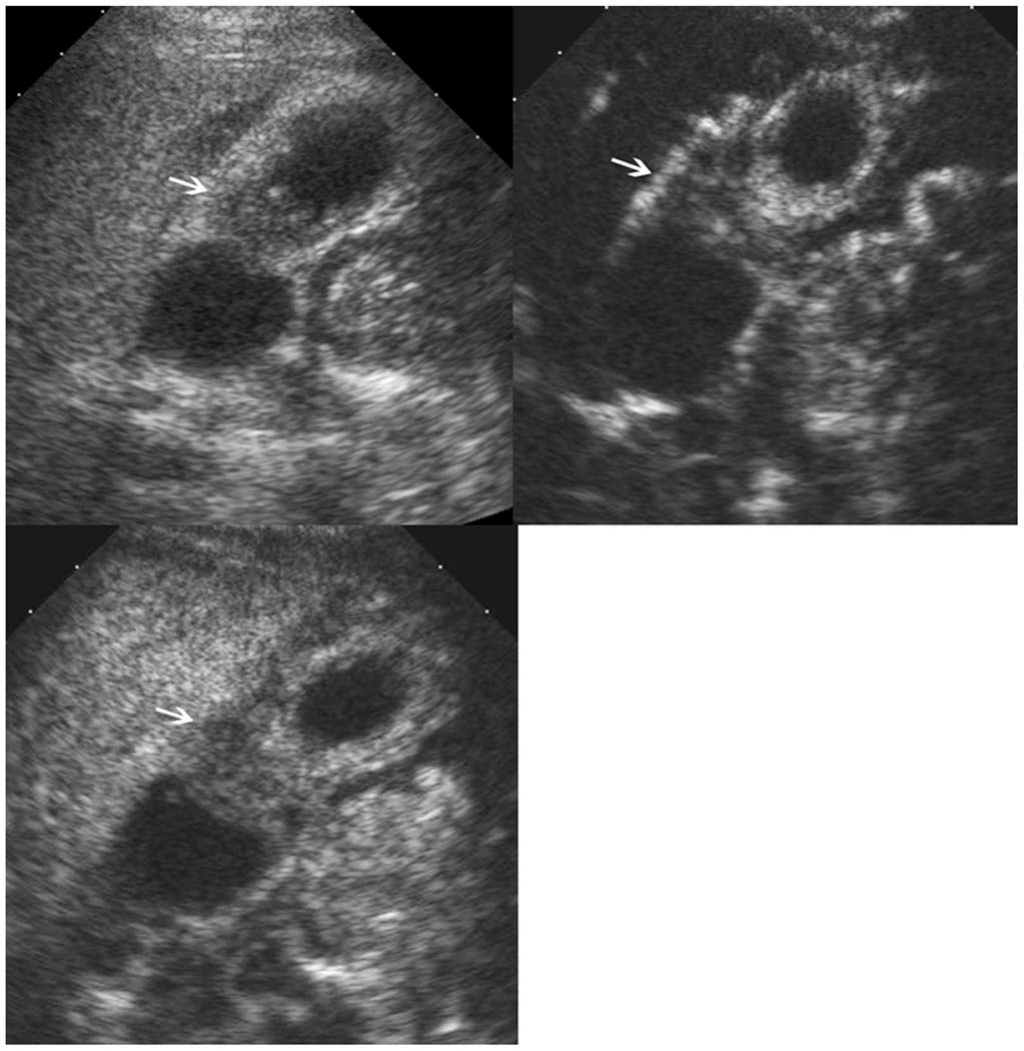
Adenomyomatosis in gallbladder. Upper left, conventional ultrasound shows a slight hypoechoic mass (arrow) in the gallbladder; Upper right, the lesion (arrow) shows inhomogeneous hyper-enhancement during the arterial phase; Lower left, the lesion (arrow) shows hypo-enhancement during the venous phase.

**Figure 6:**
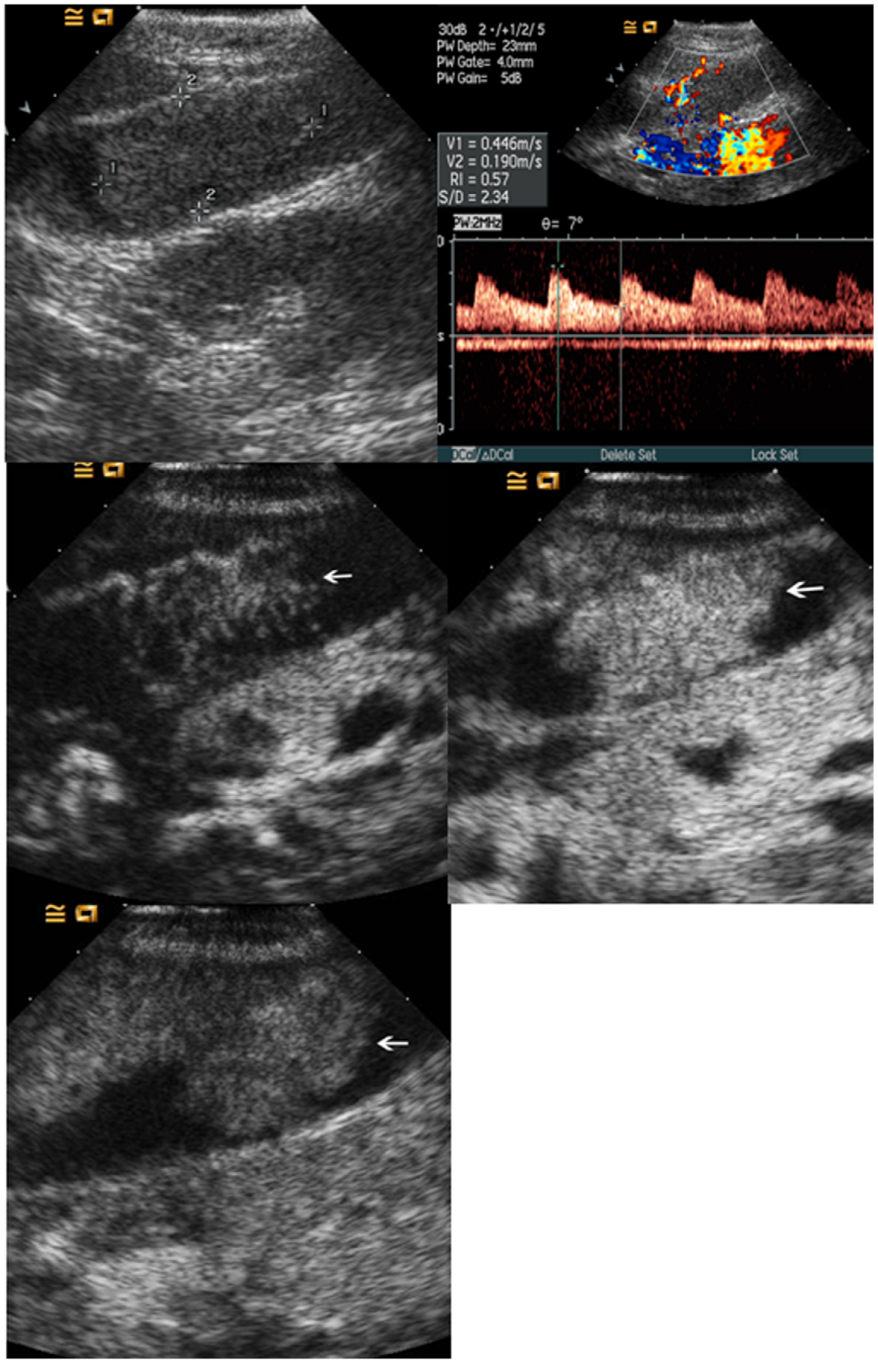
Adenoma in gallbladder. Upper left, conventional ultrasound shows an isoechoic mass (calipers) in the gallbladder; Upper right, pulsatile arterial blood flow is detected in the lesion; Middle left, the lesion (arrow) shows linear blood vessels during the arterial phase; Middle right, the lesion (arrow) shows hyper-enhancement during the arterial phase; Lower left, the lesion (arrow) shows iso-enhancement during the venous phase.

**Figure 7:**
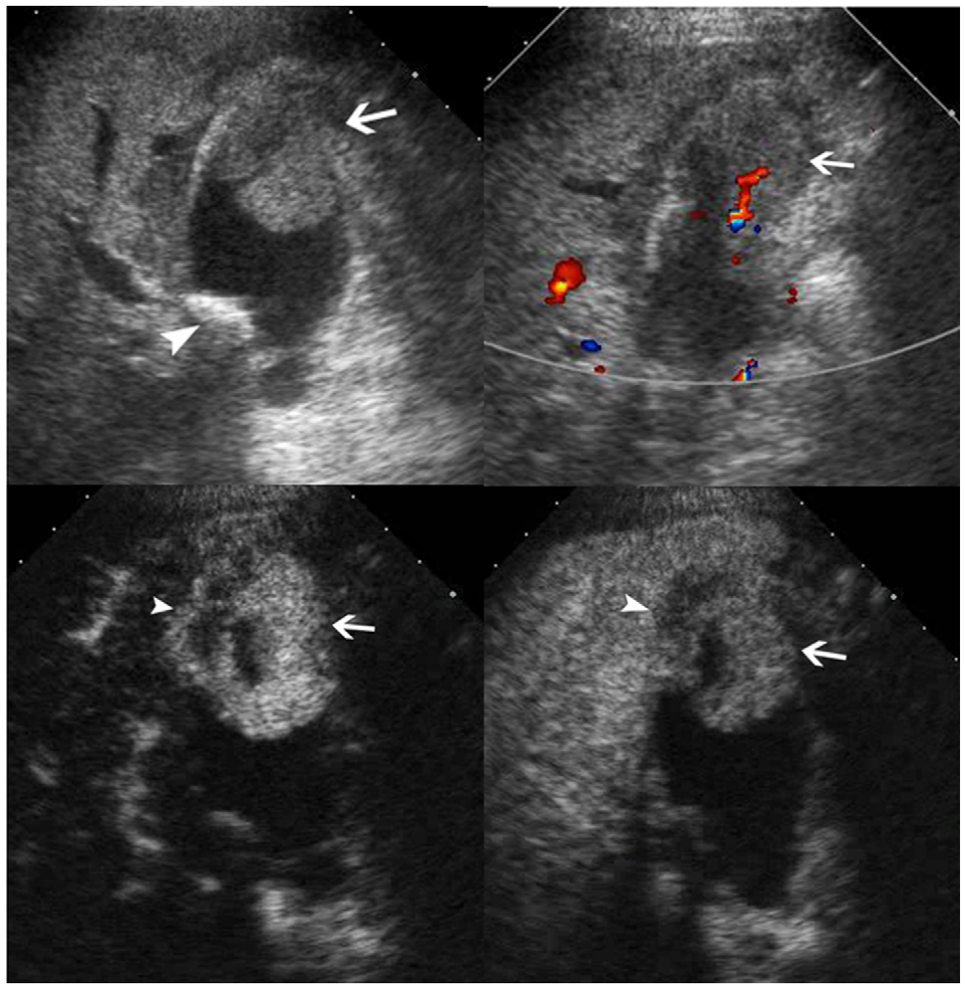
Adenocarcinoma in gallbladder. Upper left, conventional ultrasound shows an isoechoic mass (arrow) and a stone (arrowhead) in the gallbladder; Upper right, color flow imaging shows intralesional vascularity (arrow); Lower left, the lesion (arrow) shows hyper-enhancement during the arterial phase and the infiltration (arrowhead) to the adjacent liver is seen; Lower right, the lesion (arrow) shows hypo-enhancement during the venous phase and the infiltration (arrowhead) to the adjacent liver is seen.

Baseline US and CEUS were performed by radiologists with more than five years’ experience in liver US and at least two years’ experience with CEUS in liver. The US examination was performed according to the following standardized protocol, assessed at a consensus meeting prior to the study. Before the start of the study, several tentative cases with GB diseases received CEUS using different US systems in each center according to the standardized protocol. The baseline US and CEUS images were send to the coordinators for check to ensure that the CEUS procedures were standardized and the quality of the data met the requirement. After the start of the study, a medium–term inspection was carried out that the coordinators visited all the centers and reviewed the relevant data to guarantee the standardization of the procedures.

**Table 3:** The independent variables associated with the GB benign diseases by multiple logistic regression analysis.

	B	Odds ratio	95% CI	*P* Value
Age (yrs)	−0.156	0.856	0.795–0.922	<0.001
Intralesional vessels on CEUS*				0.002
Branched vs. Dotted	−23.158	0.000	0.000–NA	0.999
Linear vs. Dotted	−4.314	0.013	0.001–0.153	0.001
Wash-out time (sec)	0.064	1.066	1.025–1.110	0.002
Wall intactness on CEUS^#^	−2.945	0.053	0.012–0.230	<0.001
Constant	14.066	1285078.5	NA	<0.001

* The reference group is dotted intralesional vessels. # The reference group is intact wall on CEUS.

**Table 4:** ROC analyses of the independent variables in differentiating malignant from benign GB diseases.

	Az*	Cut-off value	Sensitivity	Specificity
Age	0.805 (0.746–0.863)	53.5 y	92.2%	59.6%
Intralesional vessels on CEUS	0.813 (0.751–0.875)	dotted	59.8%	98.0%
Wash-out time	0.669 (0.585–0.753)	36.5 s	74.8%	49%
Wall intactness on CEUS	0.857 (0.786–0.928)	intact wall	78.4%	92.9%

* Numbers in the parenthesis indicate 95% confidence interval.

All the patients underwent fasting at least 8 h before US examination. Each patient underwent a complete examination of the GB and the adjacent liver under baseline US before CEUS. The thickness of the GB wall and the maximal diameter of the GB lesions were measured on US. A second-generation blood pool agent, BR1 (SonoVue), consisting of phospholipid-stabilized shell microbubbles filled with sulfur hexafluoride gas, was used in this study. In each case, a dose of 1.5 to 2.4 mL SonoVue was administered via the antecubital vein in a bolus fashion (within 1–2 s), followed by a flush of 5 mL of 0.9% normal saline by using a 20-gauge cannula. The doses for the different systems were recommended by the manufacturers, and generally same dose was used for the same machine.

**Table 5:** The advantage of CEUS measures over conventional US in the diagnosis of GB diseases.

	CEUS	Conventional US
Depiction of the lesion features		
Width of the fundus	++	+
Destruction of the wall	+++	+
Infiltration to the adjacent liver tissue	+++	+
Metastasis to the liver	+++	+
Macro-vascularity	+++	+
Micro-vascularity	+++	−
Dynamic perfusion	+++	−
Diagnosis		
Differentiating chronic cholecystitis with thickened wall from GB carcinoma with thickened wall	+++	+
Differentiating motionless sludge from GB cancer	++++	+
Differentiating polyps or adenoma from polypoid GB cancer	+++	++
Detecting GB cancer when more than two types of GB diseases are present or the lesions fill the GB	+++	+

“+” means positive and “−” means negative.

CEUS was performed after baseline US. The target lesion was placed in the center of the screen and the transducer was kept in a stable position. The imaging mode was changed to CEUS, and the MI settings were adjusted to provide sufficient tissue cancellation with the maintenance of adequate depth penetration. Focus was positioned just below the bottom of the lesion and maintained the same position during the examination. A stopwatch was started at the time of SonoVue administration. CEUS images were recorded continuously for a period of 120 s immediately after the injection, without any change in the machine settings. After 120 s, the transducer was moved to scan the adjacent liver tissue. The vascular phases of the GB are different from those of the liver because the blood supply is provided entirely by the cystic artery and not by portal vein branches. The arterial phase is followed by the venous phase [10]. The timing of the CEUS phases were as follows: arterial phase (<30 s) and venous phase (31–120 s) [10]. After 120 s, the transducer was moved to scan the liver to exclude the liver infiltration or liver metastasis. Baseline US images and CEUS cine clips were stored digitally on the hard disks in the imaging systems, and were transferred to a personal computer for subsequent analysis.

### Image Analysis

Baseline US images and CEUS cine clips were evaluated by two experienced investigators with consensus. Both investigators had more than 4 years’ experience in GB CEUS. Before the image analysis, several tentative cases were allocated to the 2 investigators for evaluation and discussion unless a high interobserver agreement was achieved. The baseline US and CEUS images were randomized for presentation so that the GB diseases were not grouped by diagnosis, and any identifying information (i.e. the site number, the case sequence number, name, sex and age of each patient) was concealed. The investigators were anonymous to the final diagnoses of the GB diseases and were blind to the clinical relevant data such as laboratory tests and other imaging results. The baseline US and CEUS were analyzed by the same investigators with the baseline US images evaluated in advance of CEUS images. The US features of the gallbladder lesions were documented: echogenicity (hyperechoic, isoechoic, hypoechoic, mixed); location (bottom, body, neck, whole GB); morphological type (polypoid type, thickened-wall type, mass-forming type) (Fig. 1); feature of the lesion bottom (narrow, broad, not applicable). Color Doppler imaging was then used to assess the lesion vascularity. The vascularity was classified as abundant, scarce, or none.

On CEUS, the contrast arrival time to the lesions, contrast arrival time to the adjacent liver parenchyma, time to peak enhancement in the lesions, and time to hypo-enhancement (washout time) were recorded. The intralesional vascularity during the arterial phase was categorized into branched, linear, dotted, and none (Fig. 1). The enhancement pattern was classified as homogeneous and inhomogeneous. The enhancement extent during the arterial phase and venous phase was evaluated to be hyper-, iso-, hypo-, or non-enhancement, with the reference to the adjacent normal liver tissue (Fig. 2). The GB wall under the GB lesions was divided into intact and destructed (Fig. 3). Destruction of the intactness was defined as the continuity of the gallbladder wall was incomplete. The infiltration to the adjacent liver tissue was documented to be present or absent.

### Statistical Analysis

The continuous data were expressed as mean (std). The comparison between the categorical data was tested using the *x^2^* test or Fisher exact probability test. The comparison between the continuous data was tested using the independent *t* test if normal distribution was achieved; otherwise, nonparametric Mann-Whitney U test was used. A multiple logistic regression analysis was performed to select independent variables of patient characteristics, baseline US findings, and CEUS features associated with the dependent variable (i.e., the benign nature of the lesion). The independent variables were listed in Table 1 and Table 2. In the multiple logistic regression analysis, all the independent variables were firstly included as covariates. Dummy variables were allocated to the unordered categorical variables, such as the echogenicity, location, morphological type, and intralesional vessels on CEUS, and indicator coding was performed for the unordered categorical variables [17]. For the dichotomous variables and the ordered categorical variables, dummy variables were not allocated. The forward stepwise selection method was used. The independent variables with *P* values of less than 0.05 in the multiple logistic regression analysis were selected for receiver operating characteristic (ROC) curve analysis. The diagnostic performance for each significant independent variable was expressed as the area under the ROC curve (Az). The higher the Az value, the higher the diagnostic performance [18]. The Az value ranges from 0.5 to 1. The diagnostic value is regarded as low for Az of 0.5–0.7, moderate for Az of 0.7–0.9, and high for Az greater than 0.9. The cut-off value for each significant independent variable, as well as the associated sensitivity and specificity, were obtained from the ROC analysis. A multiple logistic regression model was established using the significant independent variables and the Az value of the combination of the significant independent variables was calculated. Two-tailed *P* values of less than 0.05 were accepted as showing statistical significance. The statistical analyses were performed using the SPSS 13.0 software package (SPSS, Chicago, IL).

## Results

### Final Diagnosis

The majority of the GB diseases were histologically confirmed (n = 184, 95.8%) with specimens after surgery, and the remaining 8 cases (4.2%) were confirmed by surgery. The final diagnoses of the 192 patients included GB adenocarcinoma (n = 51), cholesterol polyp (n = 63), adenomas (n = 21), adenomyomatosis (n = 14), chronic cholecystitis (n = 31), and biliary sludge (n = 12) (Table 1).

### Comparisons between Malignant and Benign GB Diseases in Basic and Baseline US Characteristics

There were significant differences between malignant and benign GB diseases in patient age, lesion number, lesion size, and lesion location (all *P*<0.05). Malignant GB diseases were more often encountered in elder patients, patients with solitary lesion, lesions greater than 3.0 cm in diameter, and lesions affecting the whole GB (Table 1).

With regard to the morphological type, malignant GB diseases were more commonly found in lesions with thickened wall and mass-forming type (*P*<0.001) (Table 1). There were significant differences between lesion echogenicity on gray-scale US and intralesional flow signals on color Doppler imaging, with malignant GB diseases more often to be hypoechoic and to show abundant intralesional flow signals (all *P*<0.05) (Table 1).

### Comparisons between Malignant and Benign GB Diseases in CEUS Characteristics

The washout time for the malignant GB diseases was quicker than that for the benign GB diseases (*P* = 0.001) (Table 2). During the arterial phase, the intralesional blood vessels were more often to be branched or linear in the malignant GB diseases whereas dotted in the benign diseases (*P*<0.001) (Table 2). Homogeneous enhancement was more easily found in benign GB diseases whereas inhomogeneous enhancement in malignant GB diseases (*P*<0.001) (Table 2) (Figs. 4, 5, 6, 7). There was significant difference between malignant and benign GB diseases in enhancement extent during the arterial phase (*P* = 0.008) (Table 2). GB wall destruction beneath the lesions and liver infiltration were more often encountered in malignant GB diseases (both *P*<0.001) (Table 2) (Fig. 7).

### Multiple Logistic Regression Analysis to Select the Independent Variables Associated with the Nature of the GB Diseases

All the independent variables in Tables 1 and 2 were submitted to multiple logistic regression analysis. The results showed that the following independent variables were associated with the benign nature of the GB diseases, including the patient age, intralesional vessels on CEUS, contrast washout time, and wall intactness depicted on CEUS (all *P*<0.05) (Table 3).

### ROC Analyses for the Independent Variables

The results of the ROC analyses for the significant independent variables were listed in Table 4. The results showed that the patient age, intralesional vessels on CEUS, and the intactness of the GB wall depicted on CEUS yielded an Az value greater than 0.8 in each whereas the Az value for the washout time was only 0.669 (Table 4).

A multiple logistic regression model was established using the significant independent variables as following:
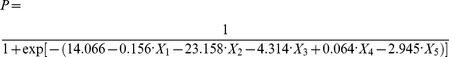
P indicated probability; *X*_1_ indicated patient age; *X*_2_ indicated intralesional vessels on CEUS (branch vs. dotted); *X*_3_ indicated intralesional vessels on CEUS (branch vs. dotted); *X*_4_ indicated washout time; *X*_5_ indicated wall intactness.

ROC analysis showed that the Az value of the combination of the four significant independent variables was 0.915 (95% confidence interval: 0.857–0.974) (*P*<0.001).

## Discussion

Conventional US is undoubtedly the first-line imaging investigation for the diagnosis of GB diseases, whereas it may face difficulty in determining the nature of the GB lesions in some complicated cases. The role of US is limited in differentiating chronic cholecystitis with thickened wall from GB carcinoma with thickened wall, differentiating motionless sludge from GB cancer, detecting GB cancer when more than two types of GB diseases are present or the lesions fill the GB. Besides that, the destruction of the GB wall beneath the lesion, and the infiltration to the adjacent liver tissue, is hard to be visible by conventional US, whereas these two features are highly suggestive of malignancy [3], [4], [5] (Table 5).

The successful use of CEUS in other organs such as liver, kidney, and pancreas has prompted the use of CEUS in GB [13], [15], [19]. Unfortunately, its usefulness in GB is controversial. Kato et al [20] found that there was no difference between cholesterol polyp and polypoid GB cancer in either enhancement pattern or the duration of the enhancement on CEUS. Inoue et al [5] concluded that vascular pattern may simply reflect the size of the lesion and the usefulness of CEUS in diagnosing GB lesions may be limited. In the 2011 non-liver CEUS guideline, the panel experts agreed that the differentiation between benign and malignant GB lesions is mainly determined by clinical features and size and enlargement of polyps to >10 mm is an indication for cholecystectomy. According to the guideline, more sophisticated classification of the vascular and enhancement pattern of GB lesions on CEUS has not been introduced so far in the clinical routine and CEUS currently has no role in differentiating benign from malignant GB polyps [10].

On the other hand, Hirooka et al [3] found that GB cancer showed enhancement, whereas cholesterol polyp showed non-enhancement using endoscopic US and air-filled microspheres created by sonication of a 5% solution of human serum albumin. The accuracy of depth of tumor invasion for endoscopic US was 78.6% versus 92.9% for contrast-enhanced endoscopic US. The authors concluded that contrast-enhanced endoscopic US is useful in the diagnosis of GB lesions. Numata et al [11] proposed tumor enhancement and tortuous-type tumor vessels on CEUS as diagnostic criteria for GB carcinomas, and the sensitivity, specificity, and accuracy of CEUS were 75%, 100%, and 91%, respectively. The authors concluded that evaluation of tumor vessels on CEUS may be useful for differentiating GB carcinoma from other polypoid gallbladder lesions [11]. Hattori et al [21] reported that when diffuse type and branched type tumor vessels were considered as indicative of cancer, the sensitivity, specificity, and accuracy were 100%, 76.9%, and 84.5%, respectively. Tusji et al [14] indicated that branched tumor vessels were possibly the characteristic of GB malignancy. Xie et al [4] found that destruction of GB wall intactness on CEUS yielded the highest capability in differential diagnosis, with sensitivity and specificity of 84.8% and 100% respectively.

To further evaluate whether CEUS is useful in the diagnosis of GB diseases, this multicenter study was carried out to assess the performance of CEUS in the diagnosis of GB diseases and find out the diagnostic criteria for GB malignancy. Multiple logistic regression analysis showed that the following independent variables were associated with the benign nature of the GB diseases, including the patient age, intralesional vessels on CEUS, contrast washout time, and wall intactness depicted on CEUS. The ROC analyses showed that the patient age, intralesional vessels on CEUS, and GB wall intactness on CEUS achieved the highest diagnostic performance in differentiating malignant from benign GB diseases, with Az value ranged from 0.805 to 0.857 respectively. The sensitivity and specificity were 59.8%–92.2% and 49%–98.0% respectively. The study was consistent with the previous studies that branched or linear intralesional vessels on CEUS and the destruction of GB wall were possibly the characteristics of GB malignancy and might be the diagnostic criteria of GB malignancy on CEUS [4], [11], [14], [21] (Table 5).

In this study, the majority of both GB carcinomas (88.2%, 45/51) and benign GB diseases (78.7%, 111/141) appeared as hyper-enhancing in the arterial phase, therefore, hyper-enhancing in the arterial phase and washout in the venous phase is not a clue for GB malignancy. On the other hand, all the carcinomas (100%, 51/51) and 87.2% (123/141) of benign GB diseases appeared as hypo-enhancing in the venous phase, thus hypo-enhancing in the venous phase can not be used for distinction between malignant and benign GB diseases, which is not like what happened in the diagnosis of liver malignancy [9]. However, the contrast washout time for the malignant and benign GB showed significant difference (41.4 s versus 58.2 s) and the ROC analysis also showed that the Az value did achieve statistical significance.

Despite of the controversies over the usefulness of CEUS in GB as mentioned above, nearly all the investigators agreed that CEUS is clearly superior to the other techniques in discriminating biliary sludge from other GB lesions [4], [5], [14]. The absence of enhancement in biliary sludge allows differentiation from a tumor, which enhances, in almost all cases on CEUS [4], [5], [10], [14]. In the cases of GB malignancy, the role of CEUS to detect infiltration of the surrounding liver parenchyma and to exclude liver metastases has been endorsed [10], [14]. In the case of acute cholecystitis, the detection or exclusion of abscess formation in the surrounding liver parenchyma is important and can be performed with CEUS, although published evidence is sparse so far. Interruption of the gallbladder wall suggests perforation, which can be confirmed by the absence of enhancement of the perforated wall [10] (Table 5).

In addition to the CEUS feature, this study found that the patient age is also a major factor for differential diagnosis between benign and malignant GB diseases. Patient age less than 53.5 yrs was more often observed in benign GB diseases. Several studies also have proven that gallbladder cancer increases with age and patient age >60 yrs is a major risk factor for malignant GB diseases [22], [23].

The primary goal of this study was to evaluate the diagnostic performance of CEUS in the differential diagnosis between malignant and benign GB diseases, thus the comparisons between baseline US and CEUS were not performed. Future studies in this regard are necessary to determine the real impact of CEUS in clinical practice. In addition, a prospective study is needed to confirm the accuracy of the proposed diagnostic criteria for GB diseases.

In conclusion, the multicenter experience of CEUS in GB diseases confirmed that CEUS is valuable in the differential diagnosis between malignant and benign GB diseases. The branched or linear intralesional vessels on CEUS and destruction of GB wall are the CEUS features highly suggestive of GB malignancy and the patient age >53.5 yrs is also a clue for GB malignancy.
